# Community Parameters and Genome-Wide RAD-Seq Loci of *Ceratothoa oestroides* Imply Its Transfer between Farmed European Sea Bass and Wild Farm-Aggregating Fish

**DOI:** 10.3390/pathogens10020100

**Published:** 2021-01-21

**Authors:** Ivona Mladineo, Jerko Hrabar, Željka Trumbić, Tereza Manousaki, Alexandros Tsakogiannis, John B. Taggart, Costas S. Tsigenopoulos

**Affiliations:** 1 Institute of Oceanography and Fisheries, Laboratory of Aquaculture, 21000 Split, Croatia; hrabar@izor.hr; 2 Institute of Parasitology, Biology Centre of Czech Academy of Science, 37005 Ceske Budejovice, Czech Republic; 3 University Department of Marine Studies, University of Split, 21000 Split, Croatia; ztrumbic@unist.hr; 4 Hellenic Centre for Marine Research, Institute of Marine Biology, Biotechnology and Aquaculture (IMBBC), 71003 Heraklion, Greece; tereza@hcmr.gr (T.M.); tsakalex@hcmr.gr (A.T.); tsigeno@hcmr.gr (C.S.T.); 5 Institute of Aquaculture, Faculty of Natural Sciences, University of Stirling, Stirling FK9 4LA, UK; john.taggart.ioa@gmail.com

**Keywords:** *Ceratothoa oestroides*, *Dicentrarchus labrax*, mean abundance, mean intensity, parasite transfer, prevalence, RAD-Seq

## Abstract

Wild fish assemblages that aggregate within commercial marine aquaculture sites for feeding and shelter have been considered as a primary source of pathogenic parasites vectored to farmed fish maintained in net pens at an elevated density. In order to evaluate whether *Ceratothoa oestroides* (Isopoda, Cymothoidae), a generalist and pestilent isopod that is frequently found in Adriatic and Greek stocks of farmed European sea bass (*Dicentrarchus labrax*), transfers between wild and farmed fish, a RAD-Seq (restriction-site-associated DNA sequencing)-mediated genetic screening approach was employed. The double-digest RAD-Seq of 310 *C. oestroides* specimens collected from farmed European sea bass (138) and different wild farm-aggregating fish (172) identified 313 robust SNPs that evidenced a close genetic relatedness between the “wild” and “farmed” genotypes. ddRAD-Seq proved to be an effective method for detecting the discrete genetic structuring of *C. oestroides* and genotype intermixing between two populations. The parasite prevalence in the farmed sea bass was 1.02%, with a mean intensity of 2.0 and mean abundance of 0.02, while in the wild fish, the prevalence was 8.1%; the mean intensity, 1.81; and the mean abundance, 0.15. Such differences are likely a consequence of human interventions during the farmed fish’s rearing cycle that, nevertheless, did not affect the transfer of *C. oestroides*.

## 1. Introduction

The isopod *Ceratothoa oestroides* (Cymothoidea, Isopoda) is one of the most detrimental ectoparasites for the European sea bass (*Dicentrarchus labrax*) fingerlings reared in in-shore aquaculture facilities in endemic marine Adriatic and Greek areas [1]. This generalist species attaches to the fish tongue and grows throughout the oro-pharyngeal cavity, becoming sedentary after its last larval moult. As a protandric hermaphrodite, the larval stage (manca or pulli) firstly develops as a juvenile and then an adult male, which subsequently transitions into a juvenile and then an adult female. The adult female blocks the transition of a second co-specific individual within the same host, which consequently remains in the stage of mature male, as long as the female is present [1]. It is assumed that its haematophagous nature [2,3] causes lethargy in fish due to low tissue oxygenation, emaciation and anaemia, accompanied by weak or moderate tissue damage, that can eventually lead to host mortality [4]. Females expel infective pulli directly in the seawater column that actively swim to reach their new hosts, facilitated by water currents. Pulli can survive for up to one month in the sea depending on the water temperature and the amount of stored vitellum. *C. oestroides* has been isolated from a number of fish: bogues (*Boops boops*), annular sea bream (*Diplodus annularis*), red sea bream (*Pagellus erythrinus*), pickerel (*Spicara smaris*), gilthead sea bream (*Sparus aurata*) belonging to Sparidae, Mediterranean horse mackerel (*Trachurus mediterraneus*, Carangidae), sardines (*Sardina pilchardus*, Clupeidae), scorpionfish (*Scoarpena notata* and *S. porcus*) belonging to Scorpaenidae, golden grey mullets (*Liza aurata*, Mugilidae) and Maenidae [5,6]. Consequently, the occurrence and aggregation of various *C. oestroides*-infected wild fish species at aquaculture facilities, either for feeding or sheltering purposes, has been suggested as one of the most important causes of parasite emergence and propagation among farmed fish.

The interactions between wild and farm escapees, as well as between the aggregating wild and farmed fish species found in Mediterranean sea cages, have been the subject of research focus in the past decades, highlighting the perception of wild fish as potential sources of pathogen infections [7]. However, the discrepancy of the lack of extensive empirical knowledge in relation to epidemiological risk assessment studies, which are rarely corroborated in the field, has resulted in different fragmented case scenarios limited to particular environments and host–parasite systems [8]. For example, transfer was not detected between Adriatic wild and farmed fish populations for the monogenean *Sparicotyle chrysophrii* and *C. oestroides* using the mitochondrial DNA cytochrome oxidase I (mtDNA COI) locus [9], while it was identified for another monogenean, *Lamellodiscus echeneis* (syn. *Furnestinia echeneis*) (Monopisthocotylea, Monogenea) based on both nuclear DNA (internal transcribed spacer 1, ITS1, with partial 18S and 5.8S ribosomal DNA) and mitochondrial (mtDNA COI) marker data [10]. This indicates that environmental traits, such as oceanographic conditions (currents and water temperature), bottom type and vegetation, as well as the zootechnical practices applied at the particular location, in addition to the choice of genetic markers for inferring the structure and the sampling design, need to be carefully considered when evaluating the outcomes of such studies.

The design of an in-depth genetic study for *C. oestroides* to resolve the issue of parasite cross-infection or transfer in aquaculture epidemiology has been impeded by the lack of available genomic resources. However, the use of Genotype-By-Sequence (GBS) techniques through Next Generation Sequencing (NGS) approaches [11,12,13], which enables the concurrent identification and allele genotyping of single-nucleotide polymorphisms (SNPs), might prove to be a cost-effective solution that allows an in-depth population genetic study of this species. The advantage of GBS techniques is the independence from prior genomic information, such as existing sequence data or an assembled genome for the target species. A widely applied method for generating population-level SNP genotype data is restriction-site-associated DNA sequencing (RAD-Seq) and its subsequent variations [14,15,16]. It has been employed for non-model aquaculture species to develop genetic linkage maps, carry out genome-wide association studies (GWAS), improve reference genome assemblies and, lately, enable selection for traits of interest for aquaculture, e.g., growth, disease resistance and sex (see [13]). RADseq has also been exploited to infer genetic structures in different marine organisms [17,18,19,20,21] and also in human parasitic [22] and a handful of fish disease studies. With respect to the latter, Barría et al. [23,24] used ddRAD to determine the genetic architecture of resistance against a Gram-negative and facultative intracellular bacterium *Piscirickettsia salmonis* in two salmonids, while Carmichael et al. [25] used the methodology to identify sex-linked SNP markers in the salmon louse (*Lepeophtheirus salmonis*). In the context of differentiation among parasite populations, RAD-Seq has proved to be discriminative at fine scales. Jacobs et al. [26] used reduced-representation library sequencing (IIb-RAD sequencing) to evaluate salmon louse (*Lepeophtheirus salmonis*) populations across Ireland, Scotland and North Norway coasts, evidencing a weak global structure among them. 

Therefore, in order to resolve whether there is a transfer of *C. oestroides* between wild farm-aggregating fish and farmed fish, our study aimed to assess the isopod’s community parameters by measuring its prevalence, intensity, abundance, and genetic origins/structure in wild and farmed fish, the last by applying the versatility of RAD-Seq technology to gain novel insights into parasite epidemiology.

## 2. Results

### 2.1. Parasite Epidemiology

Higher values for all the calculated parameters were observed for wild fish compared to reared sea bass. Briefly, in the total collected sample of reared sea bass, the prevalence was 1.02%; the mean intensity, 2.0; and the mean abundance, 0.02. By contrast, the total sample of wild fish had a prevalence of 8.1 (Stern’s exact 95% confidence intervals (CI): 5.82–11.00), mean intensity of 1.81 (1.34–2.53) and mean abundance of 0.15 (bootstrap 95% CI: 0.10–0.21). However, it needs to be highlighted that the population values of the reared fish were calculated from fish at harvest, so some isopods are likely to have been removed from the population by manual cleaning during vaccination or size-sorting, or simply because of biological constraints (e.g., poor viability and shorter life spans compared to those of the fish). Detailed *Ceratothoa oestroides* population parameters for the reared sea bass and wild fish are shown in Table 1.

Few differences in prevalence were observed between sampling seasons (between July 2016 and November 2016, March 2017, and May 2017), and differences were only observed in the sample of farmed sea bass, while the difference between the left- and right-side male lodging in the buccal cavity was significant only in the bogues (Supplementary Table S1).

### 2.2. ddRAD Sequencing Output

All 310 isopods initially used in the library preparation for ddRAD sequencing showed results, but with varying quality. In total, 32,197,244 high-quality reads were successfully assigned to all individuals, with an average of 103,862 reads. Sixty-seven specimens yielded no reads. From the remaining 243 specimens (126 “wild” and 117 “farmed”), in total, 44,386 unique RAD loci were identified. For these loci, the effective per-sample coverage had a mean of 29.2x, with st. dev. = 33.4x, min. = 5.0x, and max. = 331.2x, and there were 203.2 sites per locus on average. The individuals had a range of 1 to 4338 loci (median, 1301), from the 10 to 1,000,414 reads (median, 44,056) used for building them.

To conduct all the downstream population analyses, individuals with very few data (fewer than 500 loci) were filtered out, leaving in the final dataset 146 samples, with a median number of loci of 1904 and a range of used forward reads of 7167 to 500,207.

### 2.3. Population Genetic Structure and Relatedness in C. oestroides

Based on the original 243-specimen dataset, we chose those SNPs that were available in at least 30% of the parasites RAD sequenced and that had a minor allele frequency > 0.05; 4192 of the total sites were filtered, leaving 313 variant sites (SNPs) (7.46%) in *C. oestroides*. However, following further filtering as described above, these SNPs were kept only for the final 146 individuals used for downstream analyses.

**Figure 1:**
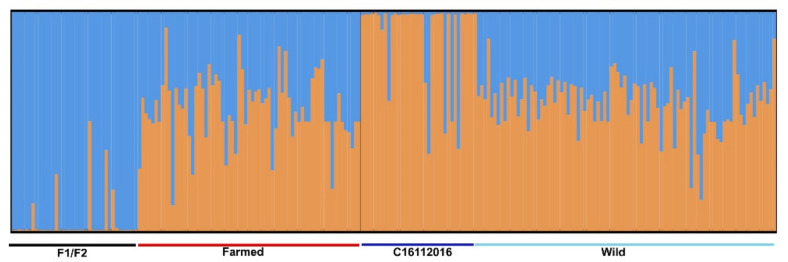
Structure analysis for *Ceratothoa oestroides* assuming two genetic clusters. Individuals are categorized according to their origin into “Farmed” and “Wild”; specimens from filial generations F1 and F2 obtained from in vivo culture of *C. oestroides* are included in “Farmed”.

The matrices built were used for population structure analysis in the species, and the outcome revealed that most specimens were a mixture of two groups/clusters (Figure 1). The only differentiation observed was due to the “F1/F2” specimens descending from known parents from European sea bass maintained in experimental tanks, and also specimens (designated “C16112016”) collected from a single wild fish (#33). In both cases, the structure was obviously affected by the sibling origins of the individuals.

**Figure 2:**
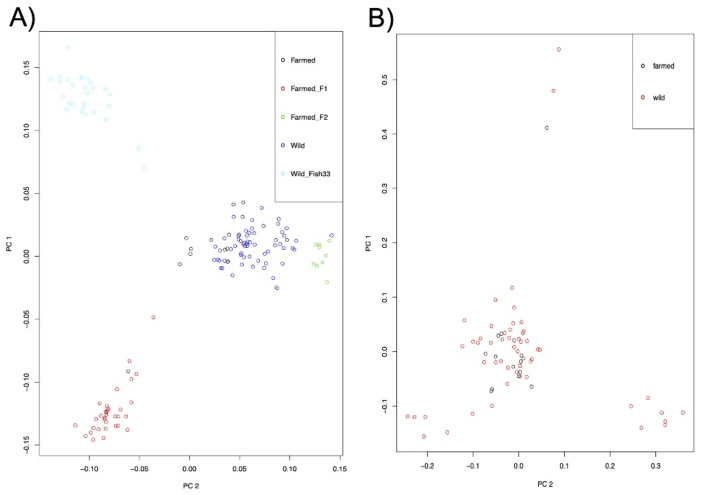
Principal component analysis (PCA) of genotypes of the isopod *Ceratothoa oestroides*: (**A**) when all collected specimens were taken into consideration, and (**B**) when sibling specimens (farmed filial generations F1 and F2 obtained from in vivo culture, and wild pulli “C16112016” collected from fish #33) were excluded. Different individual origins are shown in different colours.

Principal component analyses (PCAs) conducted with all the *C. oestroides* samples revealed a clear separation of some of the formed clusters that basically reflected the STRUCTURE matrix, i.e., all the isopods collected from the wild fish #33, as well as almost all the juveniles sampled on the 23rd of July 2016, clustered together (light blue circles; Figure 2A), belonging to the two adults and a group of pulli that were probably full-sibs. Similarly, all the genotypes belonging to the experimentally developed generation F1 (red circles in Figure 2) and F2 (green circles), as expected, grouped together. This is further supported by the top-four component weights and their pairwise plots depicted in the principal component pairs plot (Supplementary Figure S1). All the rest, i.e., the remaining farmed and wild specimens, formed a separate cluster showing high genetic relatedness (blue and black circles, Figure 2A). Repeated PCA on the samples excluding those “siblings” (F1/F2 and C16112016) further highlighted the close genetic relatedness between the rest of the farmed and wild genotypes (Figure 2B). Notably, the PCA with the excluded siblings clearly evidenced that the farmed specimens were genetically less heterogeneous (e.g., spread throughout the plot) compared to their wild counterparts (Figure 2B). None of the 313 identified SNPs showed fixed allele differences with respect to wild vs. farmed origins. 

## 3. Discussion

The population parameters of the *Ceratothoa oestroides* in the harvested sea bass evaluated in this study, i.e., the prevalence, mean intensity and mean abundance, show low values and mostly uniform patterns across the sampled seasons. By contrast, in the wild fish collected at the same aquaculture site, the parasite’s parameters highly oscillate among seasons and show considerably higher values compared to the farmed fish. Partially, this can be accounted for by the fish age susceptibility and the consequent extent of damage inflicted by *C. oestroides*. Fingerlings and juveniles suffer severe ulcers, extensive eye granulomatous lesions [27], anaemia and emaciation [28] that eventually lead to 5–20% mortality and the consequent loss of the diseased fish from the population. Survivors grow at a reduced rate, reduced by up to 20% [29,30], showing no serious pathology, except lesions mainly localized on the upper and lower jaws and the tongue [27,31], and deformities of the ventral side of the mouth [32]. The data generated in the current study indicate that, by harvest time, parasite numbers stabilise, showing no differences among seasons. In Croatian facilities, the isopod is usually extracted or “de-loused” manually from marketable fish at the sorting line before transportation to retailers. Thus, the *C. oestroides* population parameters noted herein closely depict the extent of infection at the farm site. However, this also needs cautious interpretation, as live adult isopods, although firmly attached within the buccal cavity, could be accidentally lost from the harvested fish during movement from cages to the sorting facility. Secondly, zootechnical measures implemented at the farm, such as the manual extraction of the parasites during vaccination or size-sorting, as well as the parasite’s biological constraints (e.g., viability and life span), most likely resulted in an underestimation of the population community parameters.

By contrast, wild farm-aggregating fish collected by hook and line are expected to comprise a biased subset of the population, directly influenced by *Ceratothoa* presence. Such infected, and therefore emaciated, fish are more prone to capture, likely resulting in an overestimation of the parasite prevalence from the collected samples. This scenario seems plausible also because previous *C. oestroides* epidemiological studies have concentrated on parasite parameters in fingerling and pre-commercial-size fish that, to some extent, die off, resulting in significantly higher infection values during the earlier stages of the rearing cycle. For example, the previously reported prevalence for *Ceratothoa* in Mediterranean aquaculture has varied greatly: reaching 27% in Adriatic sea bass fingerlings [33]; up to 68% in Turkish pre-commercial-size sea bass [30]; 13.7% and 20% in Greek 50–300 g-weighing sea bream and sea bass, respectively [27]; and 8.9% in Adriatic meagre (*Argyrosomus regius*) (average for two sites over seven months) [32]. Such patterns of infection values throughout the Mediterranean suggest that multiple variables affect the success of isopod infection, one amongst the most important being the feasibility of *C. oestroides*’s stage of propagate from wild farm-aggregating fish [34]. 

With the proliferative use of NGS techniques to study the genetic structures of fish parasites [26], the use of standard nuclear and mitochondrial markers (e.g., ribosomal DNA, internal transcribed spacers (ITSs), microsatellites and cytochrome oxidases (COXs)) to infer the similarity and relatedness of a parasite populating wild vs. farmed fish appears inconclusive and of low resolution in particular cases [9]. The current study represents the first application of RAD-Seq markers to infer the population structure of a parasite affecting Mediterranean fish. Based on 310 *C. oestroides* specimens that were initially included in the library preparations, we identified and scored 313 SNPs, which were used to examine the genetic relationships between wild and farmed isopod populations, and to infer whether wild farm-aggregating fish species served as a source of parasite infections for sea-cage-reared fish in a *Ceratothoa*-endemic aquaculture area. Unlike the case of the salmon lice *Lepeophtheirus salmonis* and farmed salmon (*Salmo salar*) [35], previous population genetic studies in *C. oestroides* based on mtDNA (COI) markers found significant genetic differentiation in the Adriatic Sea and indicated an absence of dispersal between wild fish (the bogue, *Boops boops*), and cage-reared gilthead sea bream (*Sparus aurata*) and European sea bass (*Dicentrarchus labrax*) [9]. The analysis of numerous, and likely neutral, SNP loci was expected to shed light and distinguish populations on a relatively small geographic scale. Our results contrast those of a previous study [9] that showed no clear genetic differentiation between isopods collected from aquaculture and wild fish using either population structure or relatedness analyses. The current data, importantly, demonstrate the ongoing parasite transfer between two fish populations. Some level of differentiation appears only as a “design bias” when isopods that are part of the same family (full-sibs; farmed F1/F2 and wild C16112016) and have therefore passed through a “genetic bottleneck” are included in the analysis. Unsurprisingly, such individuals grouped into separate clusters, but when they were excluded, the farmed and wild isopod counterparts showed a high genetic relatedness. 

Furthermore, the group of wild fish isopods proved to be more genetically heterogeneous compared to the farmed parasites, which might indicate that the former originated from a larger genetic pool found in the wild, consequently serving as the source of infection for the farmed fish. The infectivity of the pulli within cages is increased by the high density of the farmed fish population [36], but, on the other hand, it is likely to lead towards a genetic bottleneck [37]. We were not able to determine those variable markers that would distinguish wild from farmed isopod populations, which also indicates that the parasite population in the farmed fish has not been exposed to selective sweeps or suffered linkage disequilibrium, which in *L. salmonis* has been attributed to the application of chemotherapeutics [38] and local adaptation [26]. In Croatia, prophylaxis and therapy for *C. oestroides* rely on the manual removal of the parasite, rather than treatment with commercial therapeutics. The latter are employed in interventions for occasional extensive infestations and are not economically viable for most farmers [32]; therefore, no anthropogenically induced selection signature in the farmed isopod was to be expected. Our findings represent baseline data for future molecular epidemiology studies, since it is predicted that with the growth of aquaculture production and introduction of new fish species, the isopod pressure on reared stocks will increase. Consequently, disease surveillance through isopods’ molecular fingerprinting will become an essential tool for the development of integrated pest-management plans, for which the data provided herein will prove useful. 

## 4. Materials and Methods

### 4.1. Parasite Sampling and Collection of Epidemiological Data

In total, 315 specimens of *Ceratothoa oestroides* were collected from commercial-size European sea bass (*Dicentrarchus labrax*) (n = 151) farmed in a mid-Adriatic facility (43°53′24″ N 15°24′19″ E) in July (sampling period I), November 2016 (sampling period II), March (sampling period III) and May (sampling period IV) 2017. The farmed fish were checked for the presence of the isopod immediately after the harvest at the facility sorting line. The number of farmed fish was extrapolated from the total weight of the fish processed at the sorting line on the specific sampling day, and divided by the fish weight, both values acquired from the producer’s logbook.

From the same farm site, various wild, farm-aggregating fish (n = 444); blotched picarel *Spicara maena*, picarel *S. smaris*, bogues *Boops boops*, gilthead sea bream *Sparus aurata*, annular sea bream *Diplodus annularis*, sharp-snout sea bream *D. puntazzo*, common two-banded *D. vulgaris*, golden grey mullets *Liza aurata*, common pandoras *Pagellus erythrinus*, blackspot sea bream *P. bogaraveo*, axillary sea bream *P. acarne*, European sea bass *D. labrax*, and saddled sea bream *Oblada melanura* were sampled by hook and line to check for isopod infection. In total, 172 specimens of *Ceratothoa oestroides* isolated from wild hosts were collected in November 2016 (sampling period I), May (sampling period II), July (sampling period III) and October (sampling period IV) 2017. The sampling periods for the farmed and wild fish did not fully coincide because of administrative orders in place related to fishing policy, and this was considered when analysing and discussing the data. The parameters of the parasite population dynamics (prevalence, mean/median intensity and mean abundance) were calculated according to Bush et al. [39]. The Quantitative Parasitology 3.0 software [40] was used to calculate Sterne’s exact 95% confidence limits for prevalence and bootstrap 95% confidence limits (number of bootstrap replications = 2.000) for mean abundances and mean intensity. Aggregation indices were inferred through the variance-to-mean ratio and exponent of the negative binomial (k). Mood’s test was used to test the differences between median intensities. Differences in the prevalence, mean/median intensity and mean abundance of the parasite in the wild fish (total, and bogues in particular) and farmed European sea bass between the seasons and lodging sides of the male (right vs. left) were inferred with chi-square tests and bootstrap two-sample *t* tests (*p* < 0.05) (Additional File 1: Table S1).

### 4.2. Development of C. oestroides Filial Generations

Pulli (F1) hatched from known parents parasitizing European sea bass maintained in experimental tanks at the Institute of Oceanography and Fisheries, Split, were manually introduced into the buccal cavities of uninfected sea bass. The isopods (F1) developed brooding pairs, and forty days post-infection, an F2 generation hatched in experimental tanks from the respective F1 generation. DNA was extracted from the original parental pair, F1 and F2 generations, while additional DNA was extracted from unrelated but contemporary sampled isopods, with the goal of increasing the possibility of detecting polymorphic RAD markers.

### 4.3. DNA Isolation and Construction of Double-Digest Restriction-Site-Associated DNA (ddRAD) Libraries

High-molecular-weight (HMW) DNA from *C. oestroides* adults, eggs and pulli was isolated with a QIAamp DNA Mini Kit (Qiagen, Hilden, Germany) following the manufacturer’s instructions. The DNA was quantified in a 96-well plate fluorimeter using a Quant-iT™ dsDNA Assay Kit, and its integrity was assessed by electrophoresis in 1% (w/v) agarose gels.

The ddRAD library preparation protocol was based on the methodology originally reported by Peterson et al. [14] with modifications as detailed [41]. In total, 310 *C. oestroides* samples, of which 138 originated from five different parental pairs parasitizing farmed European sea bass and 172 originated from different wild farm-aggregating fish, were used in the library preparations. In order to achieve adequate coverage, three separate libraries were constructed. Only high-quality, high-molecular-weight DNA samples were used for each library preparation.

Briefly, for each library, 3 μL of the sample (21 ng of DNA in total) was digested at the same time with two high-fidelity restriction enzymes (RE): the 8-base cutter SbfI (recognition site, CCTGCA|GG) and 6-base cutter SphI (recognition site, GCATG|C) (New England Biolabs, Ipswich, Massachusetts, USA). Afterwards, barcoded adapters were individually ligated to the RE sites, and the ligated samples were combined into a single pool. The pooled sample was column-purified (MinElute PCR Purification Kit, Qiagen, Hilden, Germany) and eluted in 70 μL of EB buffer (Qiagen, Hilden, Germany). Gel electrophoresis was used to size select fragments in the range c. 400–700 bp. Following gel purification (MinElute Gel Extraction Kit, Qiagen, Hilden, Germany), the eluted size-selected template DNA (68 μL in EB buffer) was PCR amplified (15 cycles of PCR; 36 separate 12.5 μL reactions, each with 1 μL of template DNA) using a high-fidelity Taq polymerase (Q5 Hot Start High-Fidelity DNA Polymerase, New England Biolabs, Ipswich, MA, USA). The PCR reactions were combined (450 μL), purified with columns (MinElute PCR Purification Kit, Qiagen, Hilden, Germany) and eluted in EΒ buffer, resulting in ~55 μL for each library. The eluate was additionally cleaned up with an equal volume of AMPure magnetic beads (Perkin-Elmer, Ipswich, MA, USA) to remove any leftover small fragments (<200 bp) and eluted in ~22 μL of EB buffer.

Following the fluorescence-based quantification of the ddRAD libraries by Qubit (Thermo Fisher Scientific, Waltham, MA, USA), sequencing was performed on an Illumina MiSeq Next Generation Sequencer (Illumina, San Diego, CA, USA) using the Reagent kit v2 (300-cycle kit, 162 bp paired-end reads) (Illumina, San Diego, CA, USA) at the Institute of Marine Biology, Biotechnology and Aquaculture (IMBBC) of HCMR in Crete. Two MiSeq runs were performed for each library.

### 4.4. Data Analysis of ddRAD Library

The raw sequence data produced were analysed with STACKS version (v2.3) [42], which uses both reads (forward and reverse) to build one locus. Thus, SNPs that might be included in both reads are counted only once. Quality control, filtering for ambiguous barcodes and restriction sites, and demultiplexing were accomplished with the script process_radtags (options -c -q -r). The unique RAD loci and SNPs within each individual were identified with the script ustacks. To diminish potential genotypic errors, secondary reads were not used for genotype calling (option -H). Subsequently, a catalogue of loci was built for *C. oestroides* using the STACKS component cstacks with the parameter -n equal to 3 (the number of mismatches allowed between sample loci when building the catalogue). Following the catalogue construction, the loci of each individual were matched to the catalogue through sstacks. The next step included the use of gstacks to assemble and merge the second read of each pair, call variant sites and identify the genotype of each sample for each catalogue locus. The script “populations” was used to extract SNPs genotyped in at least 30% (option -r 0.3) of the individuals for population analyses. In loci with multiple SNPs, one random SNP was selected and included in the analyses (option --write_random_snp). The filtered SNPs were exported in the appropriate format for downstream analyses (i.e., structure and vcf formats).

### 4.5. C. oestroides Genetic Structure

The software STRUCTURE 2.3.2 [43] was used to infer the most likely population structure based on the SNP data of the different populations used. The calculation was performed with an admixture model without a priori population information, using a burn-in period of 250,000 and 1,000,000 subsequent MCMC repeats for each k value between one and ten. The most likely number of groups was identified using the Dk criterion [44] and detecting the number of clusters of individuals by STRUCTURE. The admixture of populations was calculated such that all individuals were assigned to each of the identified ancestral gene pools. Afterwards, their respective proportions of membership were computed.

The package SNPRelate [45] running within the R statistical environment was used to infer the relatedness among individuals for both parasites separately. SNP data were exported from STACKS in .vcf format and were then imported to SNPrelate. PCA of *Ceratothoa oestroides* genotypes was conducted using the function snpgdsPCA to assess the relatedness of individuals. The two most important components were used for plotting.

### 4.6. Data Deposition

The obtained sequences have been deposited in the European Nucleotide Archive, accession number PRJEB41438.

## Figures and Tables

**Table 1:** Data of the sampled Adriatic fish and infection values (prevalence, mean intensity and mean abundance) of isolated *Ceratothoa oestroides*.

Fish Species	Season	N. of Fish	Mean Length ± SD (mm)	Prevalence of Infection (%) (Range)	Mean Intensity (Range)	Mean Abundance (Confidence Intervals)	v/m
Farmed fish *Dicentrarchus labrax*		total sample (N = 55,250)	310.81 ± 8.53 *	1.02	2.00	0.02	
	I (2016)	summer (N = 14,000)	320.11 ± 6.63 *	0.7 (0.54–0.81)	2.27 (2.14–2.43)	0.01	2.50
	II (2016)	autumn (N = 16,250)	309.05 ± 4.15 *	1.1 (0.96–1.29)	2.00	0.02	1.98
	III (2017)	winter (N = 11,250)	296.74 ± 5.29 *	1.2 (1.03–1.44)	2.00	0.02	1.98
	IV (2017)	spring (N = 13,750)	300.23 ± 4.86 *	1.1 (0.98–1.34)	2.00	0.02	1.98
Total wild fish		total sample (N = 444)	22.91 ± 7.62	8.1 (5.82–11.00)	1.81 (1.34–2.53)	0.15 (0.10–0.21)	2.68
	I (2016)	winter (N = 51)	23.64 ± 6.75	9.8 (3.95–21.34)	1.60	1.60 (0.04–0.31)	1.62
	II (2016)	spring (N = 187)	19.37 ± 8.93	6.4 (3.65–10.89)	1.58 (1.17–2.00)	0.10 (0.05–0.17)	1.86
	III (2017)	summer (N = 48)	27.44 ± 2.91	10.4 (4.20–22.67)	1.40	0.15 (0.04–0.29)	1.46
	IV (2017)	autumn (N = 158)	25.53 ± 4.90	8.9 (5.28–14.47)	2.21 (1.57–3.79)	0.20 (0.10–0.40)	3.73
*Boops boops*		total sample (N = 268)	27.72 ± 4.11	9.7 (6.67–13.94)	2.04	0.20 (0.13–0.32)	2.96
		winter (N = 22)	27.30 ± 3.04	9.1 (1.64–29.07)	1.50	0.14 (0.00–0.36)	1.60
		spring (N = 62)	30.68 ± 2.83	9.7 (4.30–19.95)	2.17	0.21 (0.08–0.42)	2.21
		summer (N = 47)	27.44 ± 2.905	10.6 (4.29–23.15)	1.40	0.15 (0.04–0.30)	1.45
		autumn (N = 137)	26.53 ± 4.46	9.5 (5.32–15.60)	2.31 (1.62–4.08)	0.22 (0.11–0.43)	3.81
*Sparus aurata*		total sample (N = 20)	11.87 ± 2.38	10.0 (1.81–31.99)	1.50	0.15 (0.00–0.40)	1.60
*Dicentrarchus labrax*		total sample (N = 56)	11.48 ± 4.91	5.4 (1.48–14.90)	1.00	0.05 (0.00–0.11)	0.96

v/m: variance-to-mean ratio; * farmed sea bass mean length data were kindly provided by the facility and encompass the whole cohort, not only the fish measured on sampling dates.

## Data Availability

The data presented in this study are openly available in European Nucleotide Archive, accession number PRJEB41438.

## Supplementary Materials

The following are available online at https://www.mdpi.com/2076-0817/10/2/100/s1, Figure S1: Principal component analysis (PCA) of genotypes of all collected *Ceratothoa oestroides* specimens plot for the best four principal components in pairs. Colours correspond to the groups shown in Figure 2 (black: farmed sea bass, red: farmed sea bass filial generation 1, green: farmed sea bass filial generation 2, blue: wild farm-aggregating fish, light blue: wild farm-aggregating fish sample #33), Table S1: Comparison of differences in prevalence, mean/median intensity and mean abundance (variable II) of *Ceratothoa oestroides* in reared European sea bass (*Dicentrarchus labrax*) and *Ceratothoa oestroides* in wild fish from mid Adriatic, Croatia, by sampling seasons (variable I). * statistically significant at *p* < 0.05 (in bold), # calculation of the variable II could not be obtained using Quantitative Parasitology and collected dataset.
