# Supplementary material for: Community Parameters and Genome-Wide RAD-Seq Loci of Ceratothoa oestroides Imply Its Transfer between Farmed European Sea Bass and Wild Farm-Aggregating Fish
Source: Pathogens. 2021 Jan 21;10(2):100. doi: 10.3390/pathogens10020100 (PMC7912605; doi:10.3390/pathogens10020100)
Supplement: Supplementary file 1 [file pathogens-10-00100-s001.pdf]

Table S1. Comparison of differences in prevalence, mean/median intensity and mean abundance (variable II) of *Ceratothoa oestroides* in reared European sea bass (*Dicentrarchus labrax*) and *C. oestroides* in wild fish from mid Adriatic, Croatia, by sampling seasons (variable I). * statistical significance at *P*<0.05 (in bold), # calculation of the variable II could not be obtained using Quantitative Parasitology and collected dataset.

Host	Variable I	Variable II	Test	***P*-Value**
Farmed fish (N=55,250)	sp (I-IV)	prevalence	Chi-square test	0
		median intensity	n/a	#
	sp (I-II)	prevalence	Chi-square test	0
		mean abundance	Bootstrap 2-sample t-test	#
		mean intensity	Bootstrap 2-sample t-test	1
		median intensity	Mood’s median test	1
	sp (I-III)	prevalence	Chi-square test	0
		mean abundance	Bootstrap 2-sample t-test	#
		mean intensity	Bootstrap 2-sample t-test	1
		median intensity	Mood’s median test	1
	sp (I-IV)	prevalence	Chi-square test	0
		mean abundance	Bootstrap 2-sample t-test	#
		mean intensity	Bootstrap 2-sample t-test	1
		median intensity	Mood’s median test	1
	sp (II-III)	prevalence	Chi-square test	0
		mean abundance	Bootstrap 2-sample t-test	#
		mean intensity	Bootstrap 2-sample t-test	1
		median intensity	Mood’s median test	1
	sp (II-IV)	prevalence	Chi-square test	0
		mean abundance	Bootstrap 2-sample t-test	#
		mean intensity	Bootstrap 2-sample t-test	1
		median intensity	Mood’s median test	1
	sp (III-IV)	prevalence	Chi-square test	0
		mean abundance	Bootstrap 2-sample t-test	#
		mean intensity	Bootstrap 2-sample t-test	1
		median intensity	Mood’s median test	1
	Position of the male (right-left)	mean abundance	Bootstrap 2-sample t-test	0
Wild fish (N=444)	seasons (I-IV)	prevalence	Chi-square test	0
		median intensity	Mood’s median test	0

sp (I-II)	prevalence	Chi-square test	0
	mean abundance	Bootstrap 2-sample t-test	0
	mean intensity	Bootstrap 2-sample t-test	0
	median intensity	Mood's median test	0
sp (I-III)	prevalence	Chi-square test	0
	mean abundance	Bootstrap 2-sample t-test	0
	mean intensity	Bootstrap 2-sample t-test	0
	median intensity	Mood's median test	1
sp (I-IV)	prevalence	Chi-square test	0
	mean abundance	Bootstrap 2-sample t-test	0
	mean intensity	Bootstrap 2-sample t-test	0
	median intensity	Mood's median test	1
sp (II-III)	prevalence	Chi-square test	0
	mean abundance	Bootstrap 2-sample t-test	0
	mean intensity	Bootstrap 2-sample t-test	0
	median intensity	Mood's median test	1
sp (II-IV)	prevalence	Chi-square test	0
	mean abundance	Bootstrap 2-sample t-test	0
	mean intensity	Bootstrap 2-sample t-test	0
	median intensity	Mood's median test	0
sp (III-IV)	prevalence	Chi-square test	0
	mean abundance	Bootstrap 2-sample t-test	0
	mean intensity	Bootstrap 2-sample t-test	0
	median intensity	Mood's median test	1
Boops boops	prevalence	Chi-square test	0
	median intensity	Mood's median test	0
Position of the male (right-left)	mean abundances	Bootstrap 2-sample t-test	0

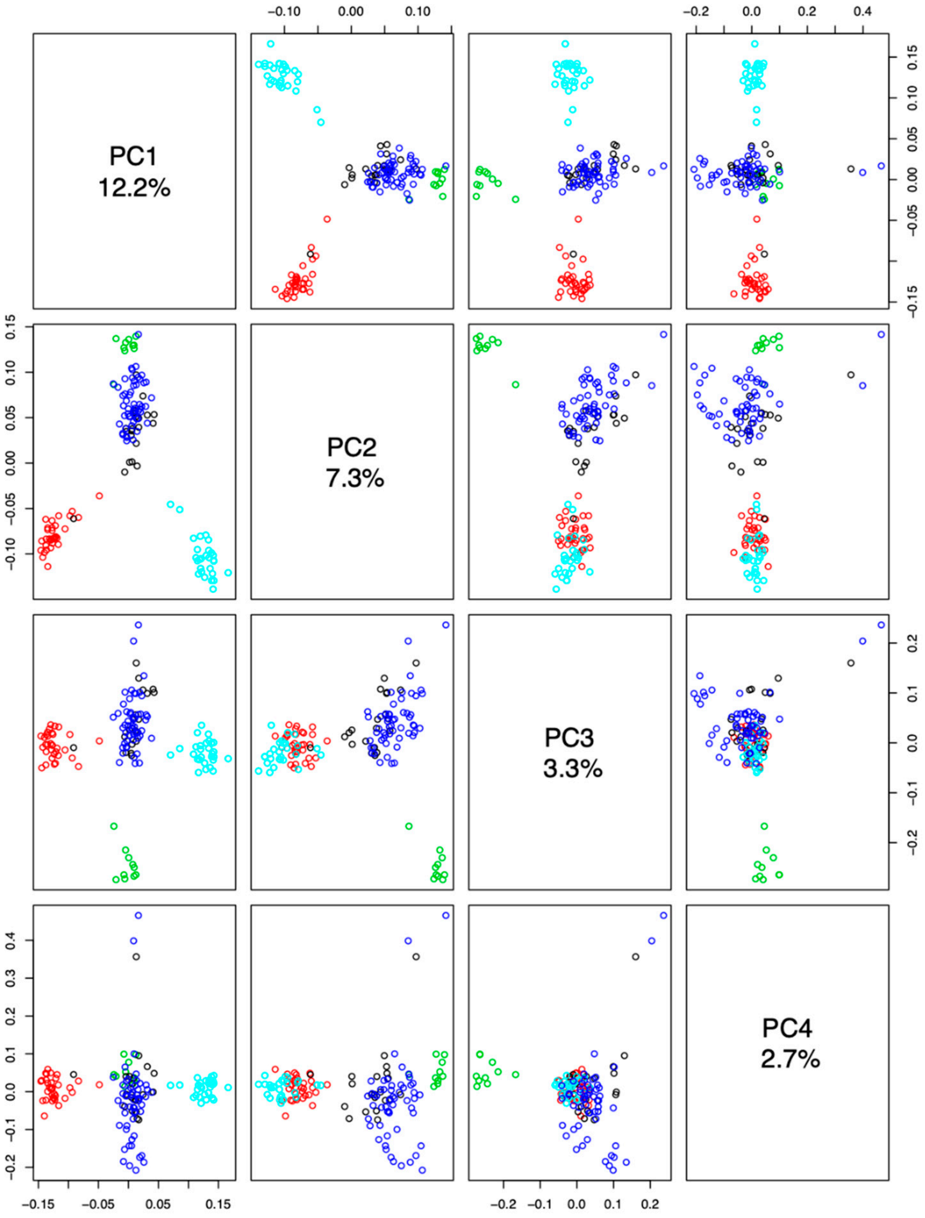

Figure S1. Principal component analysis (PCA) of genotypes of all collected *Ceratothoa oestroides* specimens plot for the best four principal components in pairs. Colours correspond to the groups shown in Figure 2 (black: farmed sea bass, red: farmed sea bass filial generation 1, green: farmed sea bass filial generation 2, blue: wild farm-aggregating fish, light blue: wild farm-aggregating fish sample #33).
